# Exploring the wound healing, anti-inflammatory, anti-pathogenic and proteomic effects of lactic acid bacteria on keratinocytes

**DOI:** 10.1038/s41598-020-68483-4

**Published:** 2020-07-14

**Authors:** Jessica Brandi, Samuele Cheri, Marcello Manfredi, Claudia Di Carlo, Virginia Vita Vanella, Federica Federici, Eleonora Bombiero, Alda Bazaj, Eleonora Rizzi, Laura Manna, Giuseppe Cornaglia, Umberto Marini, Maria Teresa Valenti, Emilio Marengo, Daniela Cecconi

**Affiliations:** 1 Department of Biotechnology, Proteomics and Mass Spectrometry Laboratory, University of Verona, Verona, Italy; 2 Department of Medicine, Section of Internal Medicine D, University of Verona, Verona, Italy; 3 Department of Translational Medicine and Center for Translational Research on Autoimmune Diseases, University of Piemonte Orientale, Novara, Italy; 4 Sintal Dietetics s.r.l., Castelnuovo Vomano, Teramo, Italy; 5 Department of Diagnostics and Public Health, University of Verona, Verona, Italy; 6 Department of Sciences and Technological Innovation, University of Piemonte Orientale, Alessandria, Italy

**Keywords:** Proteomics, Skin diseases, Mass spectrometry

## Abstract

The topical application of lactic acid bacteria (LAB) is recognized as a useful approach to improve skin health. This work aims to characterize by a multidisciplinary approach, the wound healing, anti-inflammatory, anti-pathogens and proteomic effects of six LAB lysates, belonging to the genus *Lactobacillus*. Our results demonstrated that the lysates of tested LAB stimulated the proliferation of keratinocytes, and that *L. plantarum* SGL 07 and *L. salivarius* SGL 19 accelerated the re-epithelization by inducing keratinocyte migration. The bacterial lysates also reduced the secretion of specific pro-inflammatory mediators from keratinocytes. Furthermore, viable *L. salivarius* SGL 19 and *L. fermentum* SGL 10 had anti-pathogenic effects against *S. aureus* and *S. pyogenes*, while *L. brevis* SGL 12 and *L. paracasei* SGL 04 inhibited *S. aureus* and *S. pyogenes*, respectively. The tested lactobacilli lysates also induced specific proteome modulation of the exposed keratinocytes, involving dysregulation of proteins (such as interleukin enhancer-binding factor 2 and ATP-dependent RNA helicase) and pathways (such as cytokine, NF-kB, Hedgehog, and RUNX signaling) associated with their specific wound healing and anti-inflammatory effects. This study indicates the different potential of selected lactobacilli, suggesting that they may be successfully used in the future together with conventional therapies to bring relief from skin disorders.

## Introduction

The skin, considered the largest organ of the body, is involved in a variety of functions and acts primarily as a protective barrier preventing the entry of potential pathogens. In particular, skin homeostasis is regulated by microorganisms, the so called skin microbiota, which act on keratinocytes and on their cytokine release, ensuring the good state of skin health^1^. The alteration of skin homeostasis is found in the presence of infections of wounds and during certain inflammatory diseases (for example dermatitis, acne, psoriasis).

Recent studies have suggested that the topical application of lactic acid bacteria (LAB) can improve skin health or combat disease. It has been shown that specific lactobacilli strains have a beneficial role in: wound healing process, defence against the inflammatory processes that affect skin, as well as in resistance to infections by interfering with pathogens^2–6^. For example, *Lactobacillus rhamnosus* GG lysate accelerates re-epithelialization of keratinocyte monolayers by increasing the expression of CXCL2 chemokine and of its receptor, CXCR2, that induce the keratinocyte migration^7^. Very recently, it has been also showed that *Lactobacillus rhamnosus* LR lysate improves skin barrier function in a reconstructed human epidermis model^8^, and that a multi-strain probiotic formulation, used as a topical treatment, improve the healing of infected chronic ischemic wound lesion^9^. Moreover, *Lactobacillus reuteri* ATCC-55730 exerts an anti-inflammatory effect on infected keratinocytes by reducing their transcription level of interleukin (IL)-8 and human-beta-defensin (hBD)-2^10^; while *Lactobacillus plantarum* K8 inhibits tumor necrosis factor-alpha (TNF-α) or interferon-gamma (IFN-γ) expression in keratinocytes through the lipoteichoic acid that is contained in its cell wall^11^. Furthermore, *Lactobacillus reuteri* DSM 17938 lysate exerts anti‐inflammatory effect in UVB‐stressed skin epidermal explants by reducing IL‐6 and IL‐8expression^12^. As concerning the anti-pathogenic properties of LAB, it has been demonstrated that *Lactobacillus rhamnosus* GG inhibits the *Staphylococcus aureus* infection on keratinocytes by growth inhibition and reduction of bacterial adhesion^13^; while *Lactobacillus reuteri* ATCC 55730 protects keratinocytes from *S. aureus*-induced cell death by competitive exclusion of the pathogen from its binding sites on the cells^14^. More interesting, the infection by *S. aureus* on lesional skin of patients with atopic dermatitis, was reduced and controlled by the application of a lotion containing heat-treated *Lactobacillus johnsonii* NCC 533^15^. All these results support further development of topical treatments containing LAB.

Despite these already published studies, currently a global investigation that includes the analysis of wound closure, anti-inflammatory, anti-microbial effects of different LAB has not yet been performed. In addition, their effects on keratinocytes have never been investigated from a global point of view with a proteomic approach. Here, we analysed whether the lysates of six different LAB, named *Lactobacillus paracasei* SGL 04*, Lactobacillus plantarum* SGL 07*, Lactobacillus fermentum* SGL 10, *Lactobacillus brevis* SGL 12, *Lactobacillus casei* SGL 15 and *Lactobacillus salivarius* SGL 19, may improve the wound healing process and exert anti-inflammatory and anti-pathogenic effects.

Species have been selected focusing on their potential use in preventing and treating skin disorders, considering already published experimental studies^16–21^. The goal of our study was to evaluate the effect of several strains on keratinocytes, investigating their own specific mechanism of action in order to find the best candidates for treating certain skin unhealthy conditions, based on the concept that each probiotic strain possesses unique characteristics that may influence efficacy and suitability for certain applications.

First, the promotion of wound healing process was investigated using scratch, migration, and proliferation assays. Then, following the treatment of *S. aureus* and *S. pyogenes*-exposed keratinocytes with LAB lysates, the keratinocytes viability was detected and the anti-inflammatory effect was evaluated measuring secreted cytokines by multiplex technology. In addition, the antimicrobial activity of the six tested LAB was evaluated by agar dilution method. Finally, to obtain a detailed molecular understanding of the effects induced on keratinocytes, a proteomic analysis was also carried out using high resolution mass spectrometry.

## Experimental procedures

### Keratinocyte culture

HaCaT cells (#300493, CLS Cell Lines Service, Germany), a spontaneously transformed non-tumorigenic human keratinocyte cell line, were grown in Dulbecco’s modified Eagle’s medium (DMEM, Sigma Aldrich) supplemented with 10% heat inactivated fetal bovine serum (FBS) and antibiotics (100 U/ml penicillin, 10 mg/ml streptomycin). The cells were maintained in tissue culture flasks in a humidified atmosphere of 5% CO_2_ at 37 °C.

### Bacterial cell culture

A total of 6 LAB strains, including *Lactobacillus paracasei* SGL 04 (isolated from dairy product), *Lactobacillus plantarum* SGL 07 (isolated from dairy product), *Lactobacillus fermentum* SGL 10 (isolated from dairy product), *Lactobacillus brevis* SGL 12 (isolated from local fermented cheese), *Lactobacillus casei* SGL 15 (isolated from dairy product) and *Lactobacillus salivarius* SGL 19 (isolated from aged cheese), were comprised in the present study. Before experiment, the strains stored at − 80 °C in MRS broth (Oxoid Ltd., Basingstoke, Hampshire, United Kingdom) plus 15% (v/v) glycerol, were propagated twice in MRS broth at 37 °C for 16 h.

*S. aureus* (ATCC 25923) and *S. pyogenes* (ATCC 19615) were grown in nutrient broth (NB) (Liofilchem, Teramo, Italy) and tryptic soy broth (TSB) (Liofilchem, Teramo, Italy) respectively, for 24 h at 37 °C with shaking, and washed twice in phosphate buffered saline (PBS). They were adjusted to a final optical density (OD) of 1.0 at 660 nm, followed by calculations of their colony forming units (CFUs) which corresponded to 1.5 × 10^9^ cells/ml. Both, *S. aureus* and *S. pyogenes* were heat-inactivated for 1 h at 70 °C (confirming lack of viability by cultures for 2 days), and then stored in aliquots at − 20 °C until experimental use.

### Preparation of bacterial lysates

Bacteria were pelleted at 4,500 rpm for 10 min at 4 °C, washed with 0.85% NaCl, and resuspended in 1X PBS in order to have a final concentration of 1 × 10^9^ CFU/ml. Purity of LAB cultures was checked by streaking twice on MRS agar, followed by microscopic examination. To obtain LAB lysates, 10 ml of each bacteria suspension was centrifuged, incubated for 2 h at 37 °C in lysozyme solution (10 mg/ml of enzyme in Tris–EDTA buffer pH 8.0), centrifuged, washed and resuspended in 1X PBS. LAB lysis was obtained by sonication, conducted with a Sonopulse Probe sonicator (Bandelin) in 2.5 ml of a bacteria suspension (1 × 10^9^ cells/ml). The bacterial cultures were sonicated 25 times, 15 s each with rest on ice. Lysates were filtered using a 0.22 µm-pore filter (Millipore), and protein content was determined by BCA assay (Sigma-Aldrich) using bovine serum albumin as standard.

### Keratinocytes proliferation assay

The proliferation MTS assay (Cell Titer 96 AQueous) was performed according to the manufacturer’s instructions (Promega, Madison, USA). HaCaT cells were seeded at an initial density of 5 × 10^3^ cells/well in 96 well plates, 300 μl/well. After 24 h, keratinocytes were treated for other 24 h with 90 µg of LAB lysate (to study the effect of each lysate on normal HaCaT cells); or for 12 h with 15 μl of heat-killed *S. aureus* or *S. pyogenes*, and then post-exposed for other 12 h to 90 µg of LAB lysate (to study the effect of each lysate on pathogen-stimulated HaCaT cells). Once time point was reached, cells were washed with 100 µl of 1X PBS and replaced with 100 µl of fresh media added with 20 µl of Cell Titer 96 AQueous reagent. After 2 h of incubation, the absorbance of solutions was measured at 490 nm in a TECAN Infinite 200 Pro microplate reader. The absorbance of cells in the lysate-pathogens treated cells was compared with that in their counterparts. Results were expressed as percentage of the control. The assay was conducted as three independent experiments with triplicate samples.

### Scratch wound healing assay

For scratch test, HaCaT keratinocytes were seeded on 24-well tissue culture plates. When cells reached the confluence, a scratch was introduced into the monolayer by using a sterile p200 pipette tip. Cells were then washed with 1X PBS to remove debris, and medium replaced with fresh DMEM medium plus 20% FBS (positive control samples) or DMEM medium containing 90 µg of LAB lysate. After 8, 16, 24 and 36 h scratch was monitored and documented by staining cells with crystal violet solution. Quantification of scratch closure was performed by comparing the area of the scratch according to the equation: Percentage of re-epithelialization = [(μm at t_0_ − μm at t_1_)/μm at t_0_] * 100; where t_0_ is the beginning of the experiment, t_1_ is time point at the end of evaluation. Data were obtained from three independent experiments.

### Keratinocyte migration assay

Migration of keratinocytes was determined by plating 2.5 × 10^5^ cells/well in the upper well of a 24-Transwell chamber (Invitrogen, Life Technologies Ltd) and adding 90 µg of LAB lysate under test, or the positive control (DMEM plus 20% FBS), in the lower chamber. An 8 µm pore-size permeable membrane, that allowed HaCaT cells to move toward chemo-attracting lysate, was used to separate the chambers. After 24 h or after 7 days, the membrane was washed three times with water, stained with 2 mg/ml crystal violet for 20 min, and washed five times with water before detecting cells. Non-migrating cells in the upper surface of membrane were removed, while cells adhering to the lower surface of the membrane were considered as migrated. Positive (HaCaT grown in DMEM + 20% FBS) and negative controls (untreated HaCaT, i.e. HaCaT grown in DMEM) were included to assess the technical implementation. The number of migrated cells was determined by counting the cells in three high powered fields (at 10 × magnification) and this number was subtracted from the initial number of cells seeded i.e. 2.5 × 10^5^. The experiment was performed three times with triplicate samples within each individual experiment.

### Chemokine and cytokine assay in culture supernatants

For evaluation of pro-inflammatory mediators, HaCaT keratinocytes were seeded on 24-well tissue culture plates (1 ml/well) by plating 2.5 × 10^5^ cells per well. IL-8/CXCL8, MCP-1/CCL2, RANTES/CCL5, IP-10/CXCL10, IL-1 alpha and IL-6 released into the supernatant by HaCaT cells treated for 24 h with 90 µg of LAB lysate; or exposed for 12 h to 50 μl of heat-killed *S. aureus* or *S. pyogenes* and then treated for 12 h with 90 μg of LAB lysate, were analysed by Luminex technology multiplex assay (Labospace s.r.l.) according to the manufacturer’s protocol. The analysis was conducted with quadruplicate samples for each condition.

### Antimicrobial activity of lactic acid bacteria against skin pathogens

The antagonistic activity of LAB against *S. aureus* and *S. pyogenes* was evaluated in 96-well microplates, by growing each pathogen in co-culture with each *Lactobacillus* strain. Aliquots (2 μl) of overnight cultures of LAB (10^6^, 10^7^ and 10^8^ CFU/ml), or of each LAB lysate, and pathogens (10^7^ and 10^8^ CFU/ml) were inoculated into 200 μl of fresh nutrient broth (NB) for *S. aureus* and fresh M17 medium for *S. pyogenes*. The OD of cultures at 660 nm was measured at 0, 6, 10 and 24 h. At regular intervals (0, 6, 10, and 24 h), pathogens were counted by serial dilution plate counts using respectively nutrient agar (*S. aureus*) and M17 agar (*S. pyogenes*). The assay was conducted as three independent experiments with triplicate samples.

### Label-free LC–MS/MS proteomics analyses

Identification and quantification of proteome modulation of keratinocytes treated with LAB lysates, or exposed to heat-killed *S. aureus* or *S. pyogenes* and then treated with LAB lysates, was performed as previously reported^22^. Briefly, cells were collected, washed and lysed in 1X PBS added with protease inhibitors cocktail (Roche) and 0.1% SDS. After acetone precipitation, the collected proteins were resuspended in 100 mM NH_4_HCO_3_ and quantified by BCA Protein Assay (Sigma-Aldrich, St. Louis, MO). Thus, 30 µg of extracted proteins were subjected to reduction and alkylation, and then to trypsin digestion.

Tryptic peptides were analysed by label-free LC–MS/MS, performed by using a micro-LC system (Eksigent Technologies, Dublin, USA) interfaced with a 5,600 + TripleTOF mass spectrometer (AB SCIEX, Concord, Canada). Samples were subjected first to data-dependent acquisition (DDA) analysis to generate the SWATH-MS spectral library, and then to cyclic data independent analysis (DIA), based on a 25-Da window, using three technical replicates of each sample. The MS data were acquired by Analyst TF v.1.7 (AB SCIEX); while PeakView v.1.2.0.3 and Protein Pilot v.4.2 (AB SCIEX) programs were used to generate the peak-list. Protein identification was obtained using either Protein Pilot v.4.2 (AB SCIEX) and Mascot search engines performing the database search in UniProt/Swissprot (v.2018.02.01, 42,271 sequences entries). The obtained files from the DDA acquisitions were used for the library generation using a FDR threshold of 1%. Protein quantification was performed by PeakView v.2.0 and MarkerView v.1.2. (AB SCIEX) programs by extracting from SWATH files six peptides per protein with the highest MS1 intensity, and six transitions per peptide. Peptides with FDR lower than 1.0% were exported, and up- and down-regulated proteins were selected using p-value < 0.05 and fold change > 1.5. Global relationship among samples was analysed by unsupervised principal component analysis (PCA) with MarkerView v.1.2. using normalized area of individual samples followed by auto-scaling.

### Bioinformatics analysis of proteomics data

In order to characterize the function of proteins identified by quantitative proteomics analysis, gene ontology (GO) annotation was done using the analysis tool (FunRich v3.1.3) (https://www.funrich.org/), and Reactome pathways enrichment analyses was performed as previously described^23^ by using the “Search Tool for the Retrieval of Interacting Genes/Proteins” (STRING v.11.0, https://string-db.org). In brief, differentially expressed proteins were analysed for candidate functions and pathways enrichment setting Homo sapiens as taxonomy, and p < 0.05 and gene count > 2 as cut-off point.

## Results

### Effects of lactic acid bacterial lysates on wound healing, keratinocyte migration and proliferation

**Figure 1:**
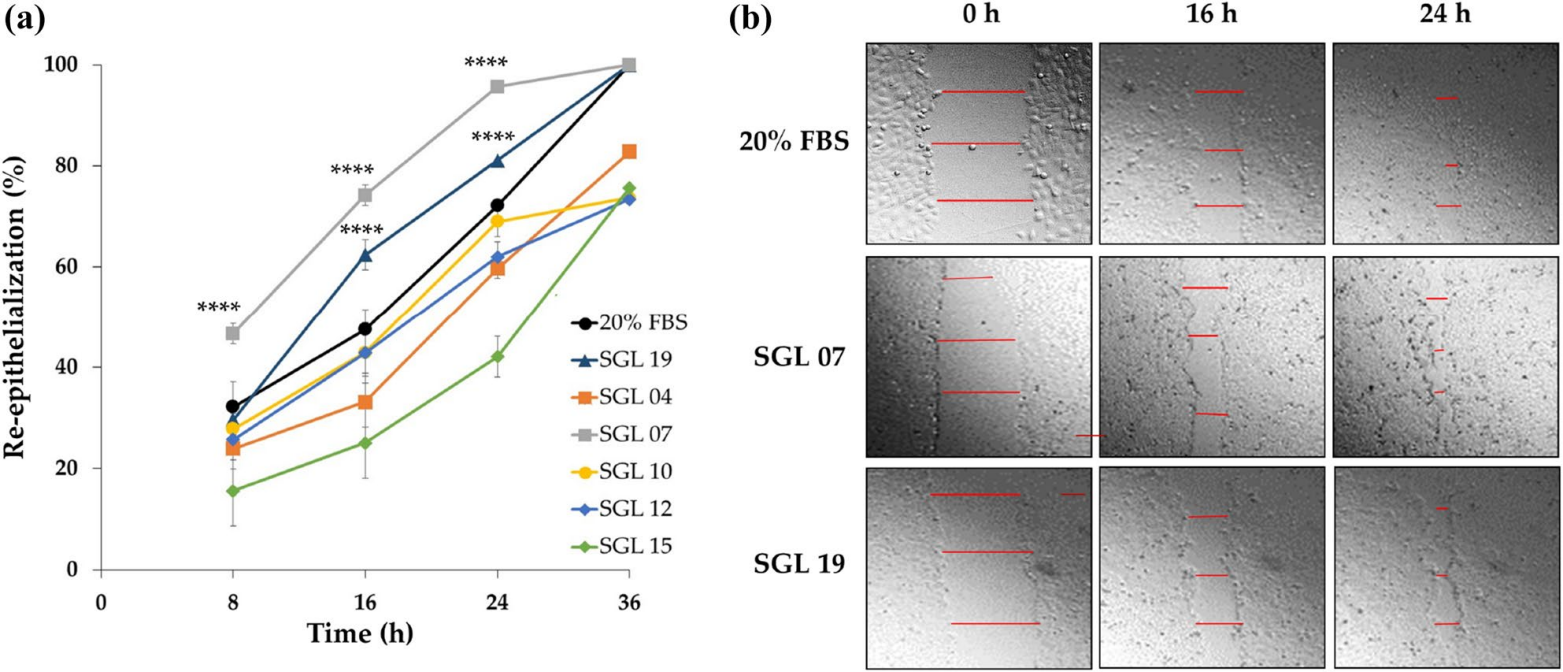
Specific LAB lysates stimulate keratinocyte re-epithelialization in vitro. (**a**) The graph shows the percentage of re-epithelialization in keratinocytes treated with/without LAB lysates for 8, 16, 24 and 36 h. Results are expressed as the mean ± SEM, ****p < 0.0001. (filled circle) positive control (20% FBS); (filled rectangle) *L. paracasei* SGL 04; (open rectangle) *L. plantarum* SGL 07; (open circle) *L. fermentum* SGL 10; (filled diamond) *L. brevis* SGL 12; (open diamond) *L. casei* SGL 15; (filled triangle) *L. salivarius* SGL 19. (**b**) Representative images (magnification, × 200) of wound closure at 0, 16 and 24 h in the presence of positive control, *L. salivarius* SGL 19 and *L. plantarum* SGL 07. Red lines indicate the distances used to calculate the % of re-epithelialization of showed representative images.

LAB lysates were prepared as described in the Methods section and used to test their wound healing properties. The scratch assay performed showed that both *L. plantarum* SGL 07 and *L. salivarius* SGL 19 significantly accelerated re-epithelialization in keratinocyte monolayers. In particular, yet at 16 h of *L. plantarum* SGL 07 exposure, 74% (p < 0.0001, n = 3) of the scratch area was re-epithelialized compared with 47% in the control monolayer; while at 24 h, 96% (p < 0.0001, n = 3) of the scratch area was closed compared with 72% of control. Accelerated scratch closure was also found when scratched cells were subjected to treatment with *L. salivarius* SGL 19, where 62% and 81% (p < 0.0001, n = 3) of the scratch area was closed at 16 and 24 h, respectively (Fig. 1a, b).

**Figure 2:**
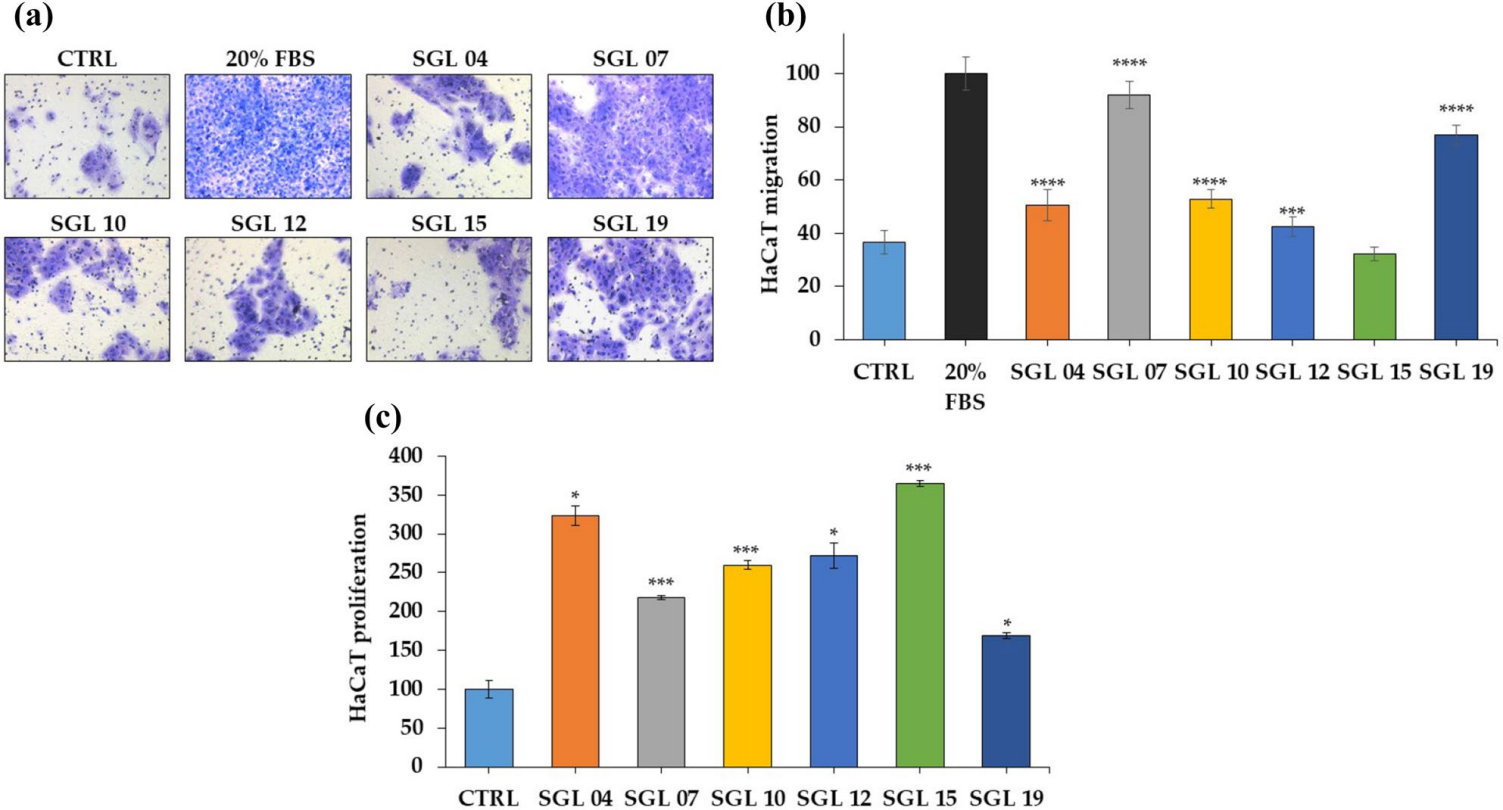
LAB lysates increased keratinocyte migration and proliferation. (**a**) Representative images of the transwell migration assay in untreated control HaCaT cells (CTRL), positive control (20% FBS) and upon treatment of keratinocytes with LAB lysates (magnification × 20). (**b**) Migration ability of keratinocytes represented as the percentage of cells penetrating the membrane over positive control. (**c**) Proliferation of keratinocytes after exposure for 24 h to LAB, measure by MTS reduction (% of control).

In contrast, all the other LAB did not stimulate monolayer re-epithelialization (Supplemental Fig. 1). To further evaluate the effects of LAB on keratinocytes, the treated cells underwent migration and/or proliferation analyses. Transwell migration assay at 7 days showed that all the LAB lysates, excepted *L. casei* SGL 15, enhance the migration capacity of HaCaT cells (Fig. 2a). Interestingly, the stronger induction of keratinocyte migration was detected for *L. plantarum* SGL 07 and for *L. salivarius* SGL 19 (which showed accelerated re-epithelialization). In particular, in HaCaT cells treated for 7 days with *L. plantarum* SGL 07 and *L. salivarius* SGL 19 there were about 92% and 77% of migrated cells respectively (both with p < 0.0001, n = 3), compared with 37% cells in untreated cells (Fig. 2b). Migration assay was performed also at 24 h showing that after one day the selected six LAB lysates had no effect on keratinocyte movement (Supplemental Fig. 2).

In addition, incubation of keratinocytes with each different LAB lysate resulted in significant increasing of proliferation relative to control culture at 24 h post-treatment (Fig. 2c). The stronger effect was that of *L. casei* SGL 15 which caused a 3.7-fold increase (p < 0.001) in the number of cells compared with the untreated control, while *L. paracasei* SGL 04, *L. brevis* SGL 12, *L. fermentum* SGL 10, *L. plantarum* SGL 07 and *L. salivarius* SGL 19 afforded stimulation of keratinocyte proliferation equal to 3.2, 2.7, 2.6, 2.2, and 1.7-fold respectively.

The ability of LAB lysates to promote proliferation of keratinocytes even after exposure to *S. aureus* or *S. pyogenes* was also investigated (Supplemental Fig. 3). Interestingly, keratinocytes incubated for 12 h with the heat-inactivated pathogen, and then for other 12 h with LAB lysates, had a higher viability than monolayers treated with the pathogen alone. The stronger effect was that of *L. plantarum* SGL 07 and *L. paracasei* SGL 04.

### Anti-inflammatory effects of lactic acid bacteria lysates

**Figure 3:**
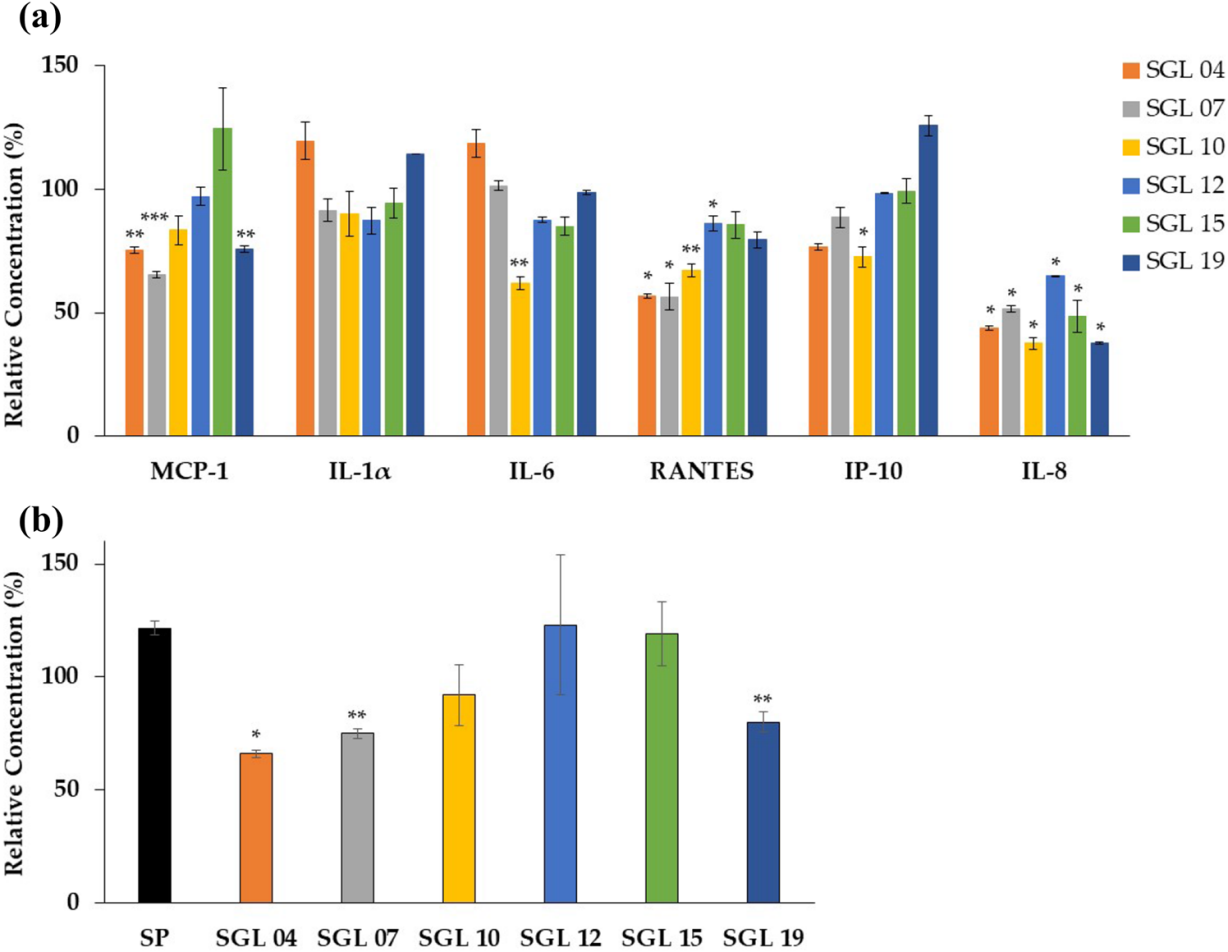
LAB lysates reduce the secretion of specific chemokines and cytokines from keratinocytes. (**a**) MCP-1, IL-1 alpha, IL-6, RANTES, IP-10 and IL-8 production in HaCaT cells exposed to different LAB measured by multiplexed ELISA assay. (**b**) MCP-1 secretion from HaCaT cells subjected to *S. pyogenes* (SP) stimulation and then exposed to different LAB. Relative concentration (%) was determined by dividing the average concentration of chemokine or cytokine in LAB-treated HaCaT cells by concentration in control (untreated HaCaT cells) × 100. Results are expressed as the mean ± SEM, ***p < 0.001, **p < 0.01, *p < 0.05.

Keratinocytes produce a plethora of pro-inflammatory chemokines and cytokines. To further investigate possible positive effects of LAB, the response of HaCaT cells to LAB lysates in terms of release of pro-inflammatory chemokines IL-8, MCP-1 RANTES, IP-10, and cytokines IL-1 alpha and IL-6, was evaluated. Noteworthy, analysis of the medium collected after 24 h exposure of keratinocyte to LAB lysates, indicated that: *L. paracasei* SGL 04 and *L. plantarum* SGL 07 decreased the release from HaCaT of MCP-1 (1.3 fold change, p = 0.0028; 1.5 fold change, p = 0.0014, respectively), RANTES (1.8 fold change, p = 0.0174; 1.8 fold change, p = 0.0241, respectively) and IL-8 (2.3 fold change, p = 0.0038; 1.9 fold change, p = 0.0050, respectively); *L. fermentum* SGL 10 down-regulated the level of IL-6 (1.6 fold change, p = 0.0070), RANTES (1.5 fold change, p = 0.0036), IP-10 (1.4 fold change, p = 0.0111) and IL-8 (2.7 fold change, p = 0.0125); *L. brevis* SGL 12 reduced the secretion of RANTES (1.2 fold change, p = 0.0252) and IL-8 (1.5 fold change, p = 0.0358); *L. casei* SGL 15 decreased the release from HaCaT of IL-8 (2.1 fold change, p = 0.0247); while *L. salivarius* SGL 19 significantly reduced the HaCaT secretion of MCP-1 (1.3 fold change, p = 0.0026) and of IL-8 (2.7 fold change, p = 0.0031) (Fig. 3a).

Moreover, the effect of LAB on chemokines and cytokine production from keratinocytes after treatment with heat-inactivated pathogens was analysed. As a consequence of *S. pyogenes* stimulation, the expression of pro-inflammatory chemokine MCP-1 was induced (1.2-fold change, p = 0.0107) as compared to HaCaT control cells. Interestingly, MCP-1 was significant decreased when keratinocytes stimulated with *S. pyogenes* were treated with: *L. paracasei* SGL 04 (1.6-fold change, p = 0.0128), *L. plantarum* SGL 07 (1.6-fold change, p = 0.0030) and *L. salivarius* SGL 19 (1.5-fold change, p = 0.0084) (Fig. 3b). No changes were instead detected for all the other chemokines and cytokines after *S. pyogenes* stimulation (Supplemental Fig. 4). Moreover, chemokines and cytokines did not reveal statistically significant modulations after stimulation for 24 h with heat-inactivated *S. aureus* probably due to the absence of real infectious conditions (Supplemental Fig. 4).

**Figure 4:**
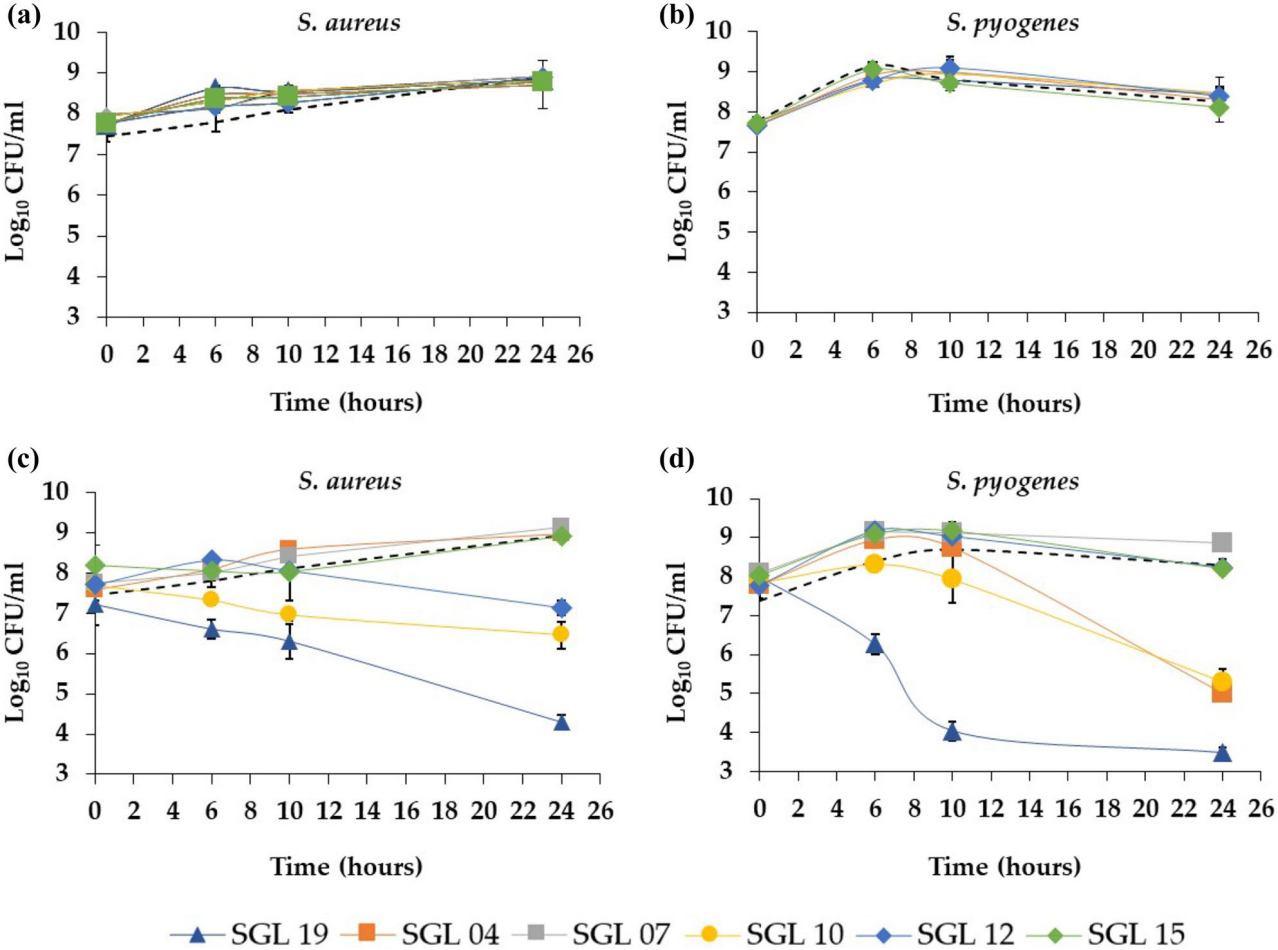
Inhibition of *S. aureus* and *S. pyogenes* growth by LAB. Antimicrobial activity of 90 µg LAB lysates on 10^7^ CFU/ml (**a**) *S. aureus* and (**b**) *S. pyogenes*, and of 10^8^ CFU/ml live LAB on 10^7^ CFU/ml (**c**) *S. aureus* and (**d**) *S. pyogenes* growth in a competition assay. Results are expressed as the mean ± SEM. (dashed lines) pathogen; (filled rectangle) *L. paracasei* SGL 04; (open rectangle) *L. plantarum* SGL 07; (open circle) *L. fermentum* SGL 10; (fiiled diamond) *L. brevis* SGL 12; (open diamond) *L. casei* SGL 15; (filled triangle) *L. salivarius* SGL 19.

### Anti-pathogenic properties of live lactic acid bacteria and lysates

To investigate whether LAB have direct effects on the growth of *S. aureus* and *S. pyogenes*, the antimicrobial activity of lysates and live LAB was analysed. The assay performed showed that lysates did not induce an inhibition of pathogen growth (Fig. 4a,b), even if viable LAB had anti-pathogenic effects. In particular, the evaluation of antimicrobial activity of viable LAB against *S. aureus*, showed a strong inhibition of *S. aureus* growth over a 24 h period in the presence of 10^8^ CFU/ml *L. salivarius* SGL 19 (4.1-log_10_ reduction, p < 0.0001), and a moderate inhibition of pathogen growth after exposure for 24 h to 10^8^ CFU/ml *L. fermentum* SGL 10 (2.5-log_10_ reduction, p < 0.0001) or *L. brevis* SGL 12 (1.8-log_10_ reduction, p < 0.0001) (Fig. 4c). Moreover, the competition assay performed for *S. pyogenes*, showed a strong inhibition of pathogen growth over a 24 h period in the presence of 10^8^ CFU/ml *L. salivarius* SGL 19 (4.8-log_10_ reduction, p = 0.0002), and a moderate inhibition of *S. pyogenes* growth after exposure to 10^8^ CFU/ml *L. paracasei* SGL 04 (3.1-log_10_ reduction, p = 0.0082) or *L. fermentum* SGL 10 (3-log_10_ reduction, p = 0.0124) (Fig. 4d). These effects were specific to the live LAB strains, because the lysates had no effects on the growth of *S. aureus* and *S. pyogenes*.

### Proteomic modulation of biological processes and pathways in keratinocytes treated with lactic acid bacteria lysates

Furthermore, we also conducted a label-free quantitative proteomics analysis for studying alterations in protein abundances and seeking possible evidence for and understanding the mechanisms responsible for the LAB effects (Table 1) on keratinocytes.

**Table 1:** Overview of effects of LAB on HaCaT cells and pathogens.

LAB strain	Wound healing	Migration	Proliferation	Anti-inflammatory	Anti-pathogen (viable LAB)
*L. paracasei* SGL 04	=	↑	↑↑↑	↓ MCP-1, RANTES, IL-8	↓ *S. pyogenes*
*L. plantarum* SGL 07	↑	↑↑	↑	↓ MCP-1, RANTES, IL-8	=
*L. fermentum* SGL 10	=	↑	↑↑	↓ RANTES, IL-6, IP-10, IL-8	↓ *S. aureus, S. pyogenes*
*L. brevis* SGL 12	=	↑	↑↑	↓ RANTES, IL-8	↓ *S. aureus*
*L. casei* SGL 15	=	=	↑↑↑	↓ IL-8	=
*L. salivarius* SGL 19	↑	↑↑	↑	↓ MCP-1, IL-8	↓ *S. aureus, S. pyogenes*

**Figure 5:**
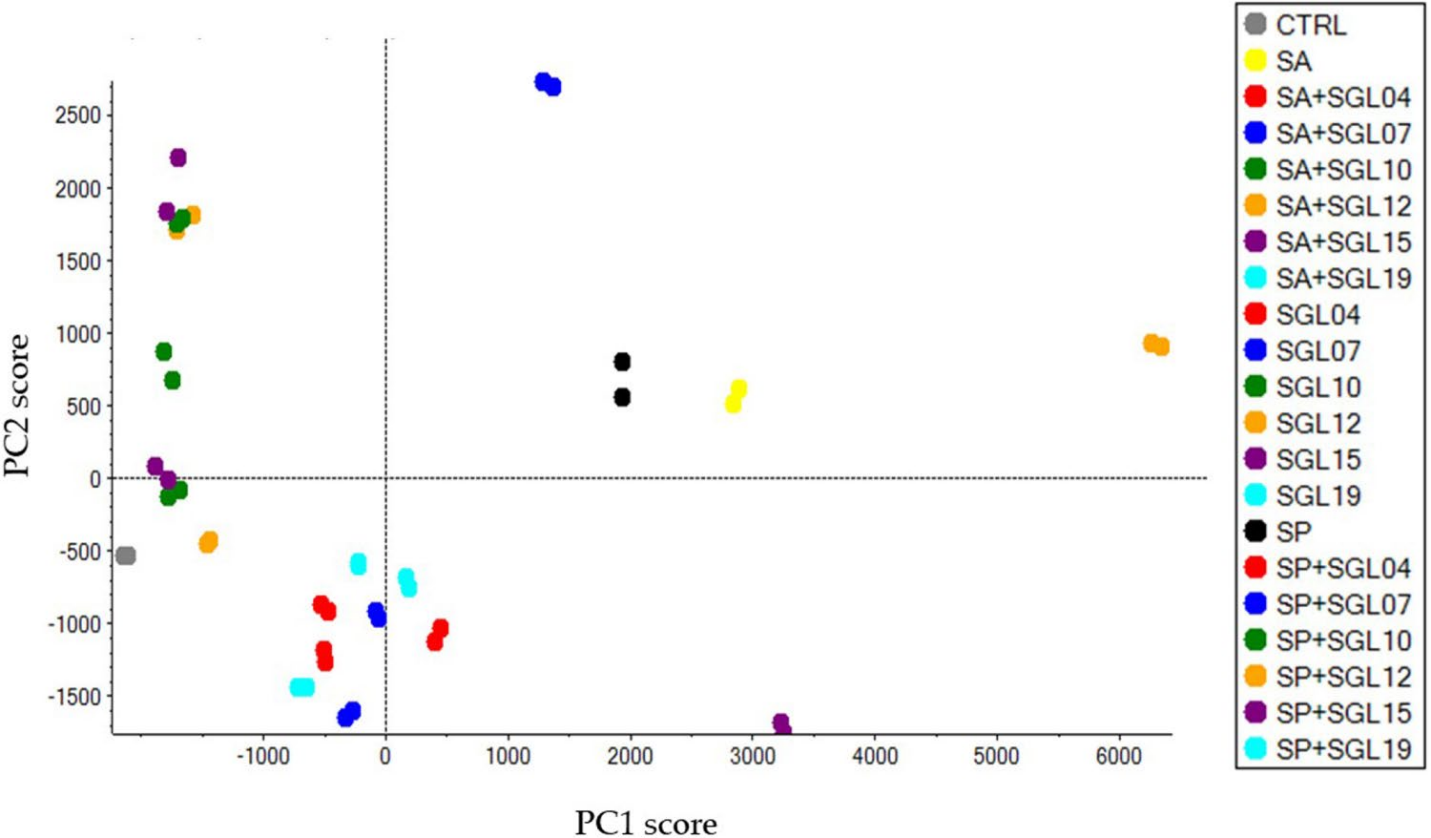
Unsupervised PCA model of protein data sets. PCA scatterplot representing HaCaT cells treated or not with the six tested LAB, with or without pathogens (SA, SP) exposure. The two principal components PC1 and PC2 were 37.1% and 13.8%, respectively.

In our proteomics experiment, two biological replicates and three technical replicates were analysed. In total, 1,154 proteins were identified, and 735 proteins quantified with a peptide confidence cut-off of 99% (FDR < 1%) (Supplemental Table 1). Variability of protein data set was examined by unsupervised PCA. The first and second components explained the 37.1% and 13.8% overall variance, respectively (Fig. 5). HaCaT cells treated with the different LAB (exposed or not to pathogens) were split into different groups which mainly correspond to the different LAB (Fig. 5). This indicates that enough natural variation exists between the treatments, increasing the confidence that interesting protein modulations can be found in these data sets.

**Figure 6:**
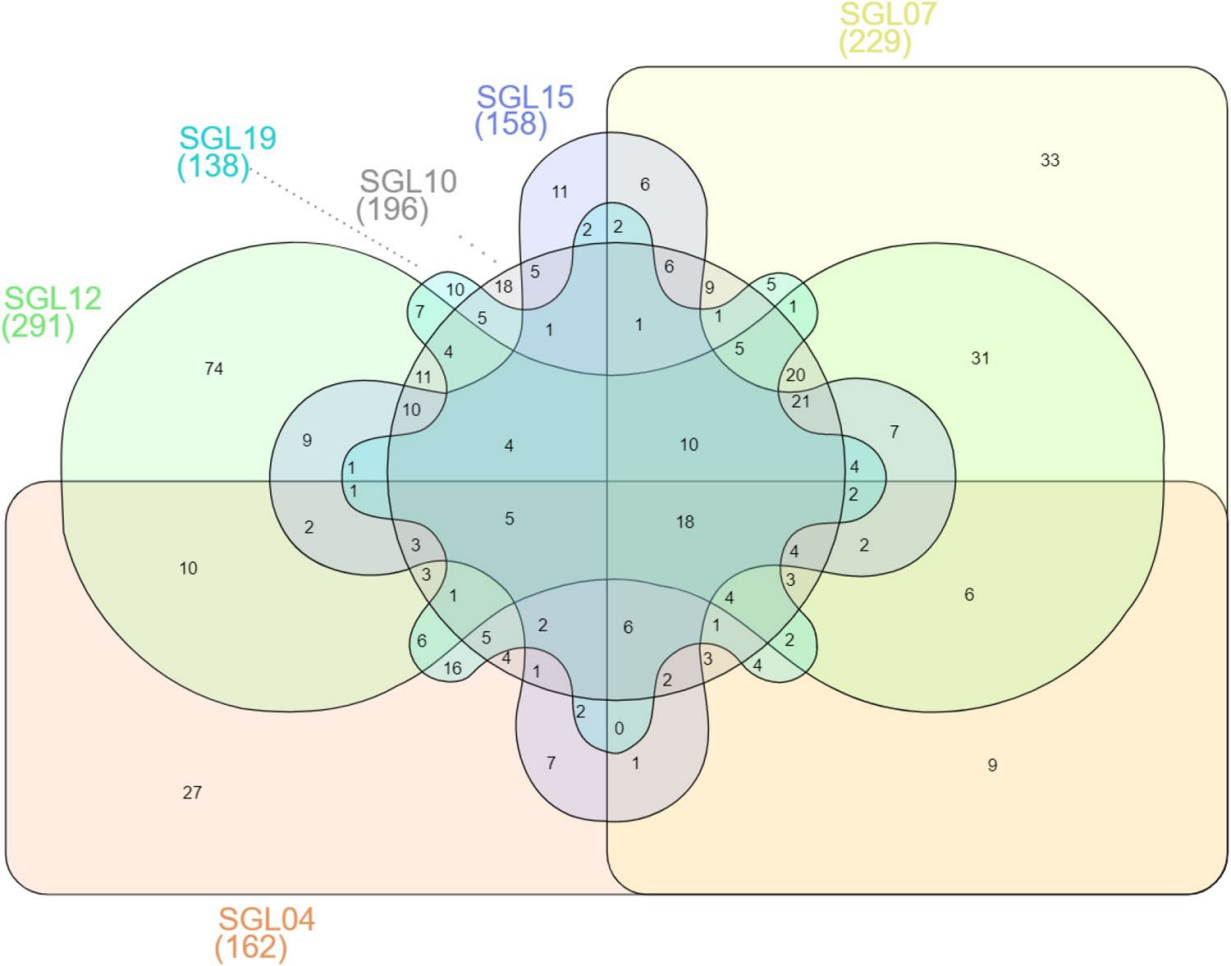
Venn Diagram summarizing deregulated proteins which were detected across all the samples. A total of 27, 33, 18, 74, 11, and 10 proteins were specifically modulated by SGL 04, SGL 07, SGL 10, SGL 12, SGL 15 and SGL 19 treatment, respectively (see Supplemental Table 3 for gene/protein name).

Differential protein expression was significant when the expression increased or decreased with a fold change of 1.5 and a p value < 0.05. A total of 162, 229, 196, 291, 158, and 138 proteins were found differentially expressed in the SGL 04, SGL 07, SGL 10, SGL 12, SGL 15 and SGL 19 treated keratinocytes compared to the control (Supplemental Table 2). Proteomic results demonstrated that there were only 18 common proteins modulated in keratinocytes regardless of the type of LAB treatment (i.e. 6PGL, ADIRF, DDX21, DDX3X, GLOD4, H2A1, HBD, ILF2, IMA1, KAD2, MOS, PTBP1, RLA1, RS29, TACD2, THIO, TRFE, and XPO2); and that there were also proteins which were found to be deregulated only by a specific lactobacillus (Supplemental Table 3). Overlapping and non-overlapping differentially expressed proteins were showed in Fig. 6.

**Figure 7:**
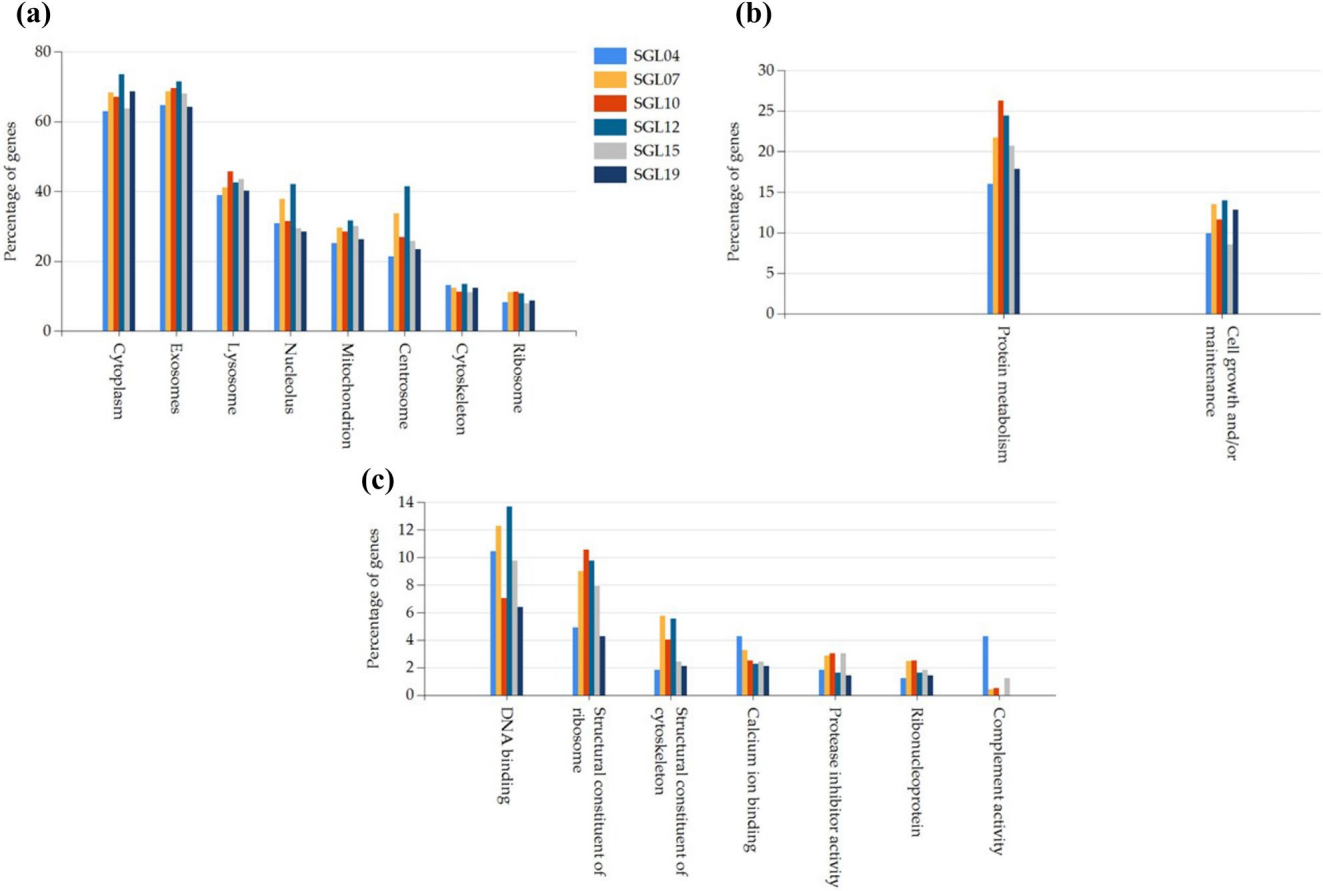
Functional enrichment analysis of deregulated proteins in keratinocytes after LAB treatments from the FunRich software for (**a**) cellular component, (**b**) biological process, and (**c**) molecular function. Bars represent the percentage of protein genes assigned to the indicated term.

The deregulated proteins by the six LAB were mapped to annotation terms within the GO cellular component, biological process and molecular function categories (Fig. 7). In particular, we found that the cellular distributions of the identified proteins were similar, and that most of the deregulated proteins (~ 60%) were either cytoplasmic and secreted as exosomes, ~ 40% were lysosomal, while ~ 30% were nuclear or mitochondrial proteins.

The up and down-regulated proteins of keratinocytes were further analysed for assignment to Reactome database (Supplemental Tables 4 and 5). The common significant enriched pathways for proteins up-regulated after LAB treatments comprised metabolism of RNA, amino acids, and proteins, as well as regulation of expression of SLITs and ROBOs which are involved in cell migration. More interesting, enriched pathway analysis revealed that the down-regulated proteins of HaCaT cells were highly associated with pathways that can be mainly related to the anti-inflammatory effect of LAB, such as: neutrophil degranulation, immune system, infectious disease, interleukin-1 signalling, cytokine signalling in immune system, and signalling by interleukins; as well as NF-kB, Hedgehog, and RUNX-related pathways (Table 2).

**Table 2:** Bioinformatics analysis of the effects of LAB lysates on keratinocytes.

	SGL 04 (# molecules; p value)	SGL 07 (# molecules; p value)	SGL 10 (# molecules; p value)	SGL 12 (# molecules; p value)	SGL 15 (# molecules; p value)	SGL 19 (# molecules; p value)
**Pathways (up-regulated proteins)**						
Developmental Biology	13; 0.0021	12; 0.0071	15; 1.58e−05	12; 0.0059	12; 0.0002	11; 0.0041
Metabolism	24; 3.73 e−05	18; 0.0059	15; 0.0118	23; 8.31 e−05	17; 0.0002	16; 0.0055
Metabolism of amino acids and derivatives	8; 0.0015	6; 0.0290	9; 3.16 e−05	7; 0.0047	7; 0.00041	10; 4.12 e−05
Metabolism of proteins	22; 0.0001	19; 0.0024	20; 2.37 e−05	20; 0.0009	16; 0.0003	19; 0.0002
Metabolism of RNA	11; 0.0010	8; 0.0325	15; 5.37 e−07	15; 4.03 e−06	12; 7.37 e−06	12; 5.79 e−05
Nonsense Mediated Decay (NMD) enhanced by EJC	5; 0.0016	none	7; 6.84 e−06	3; 0.0327	7; 2.98 e−06	6; 8.61 e−05
Nonsense Mediated Decay (NMD) independent of the EJC	5; 0.0009	3; 0.0476	7; 4.14 e−06	none	7; 2.14 e−06	6; 5.15 e−05
Peptide change elongation	4; 0.0048	3; 0.0449	6; 1.58 e−05	none	6; 7.37 e−06	5; 0.0003
Regulation of expression of SLITs and ROBOs	7; 0.0002	4; 0.0410	8; 5.98 e−06	5; 0.0046	7; 8.67 e−06	7; 5.79 e−05
**Pathways (down-regulated proteins)**						
Apoptosis	6; 0.0006	7; 0.0019	6; 0.0052	9; 0.00037	5; 0.0164	5; 0.0019
Cytokine Signaling in Immune system	7; 0.0349	17; 0.0003	13; 0.0039	21; 7.01 e−05	13; 0.0007	12; 0.0003
Dectin-1 mediated noncanonical NF-kB signaling	4; 0.0007	5; 0.0009	4; 0.0045	6; 0.0002	3; 0.0209	4; 0.0007
FCERI mediated NF-kB activation	4; 0.0015	6; 0.0006	4; 0.0096	6; 0.0009	3; 0.0310	4; 0.0014
Hedgehog ligand biogenesis	4; 0.0008	6; 0.0002	5; 0.0009	6; 0.0003	3; 0.0215	4; 0.0007
Hedgehog 'on' state	4; 0.0016	5; 0.0027	4; 0.0105	6; 0.0011	3; 0.0323	4; 0.0015
Interleukin-1 signaling	5; 0.0006	6; 0.0011	5; 0.0041	7; 0.0005	4; 0.0172	4; 0.0025
Regulation of RUNX2 expression and activity	4; 0.0010	5; 0.0015	4; 0.0067	6; 0.0005	3; 0.0251	4; 0.0009
Regulation of RUNX3 expression and activity	4; 0.0006	5; 0.0007	4; 0.0038	6; 0.0002	3; 0.0202	4; 0.0008
Signaling by Interleukins	6; 0.0200	13; 0.0006	11; 0.0023	16; 0.0002	10; 0.0017	9; 0.0008

A summary of specific pathways related to the wound healing and anti-inflammatory effects of LAB lysates, together with the number (#) of associated molecules affected and corrected *p*-values (for the complete list see Supplemental Tables 4 and 5).

Among the 18 overlapping proteins identified in all the treatments, some were associated with the anti-inflammatory effect of LAB. For example, interleukin enhancer-binding factor 2 (ILF2) downregulated by 0.41, 0.20, 0.30, 0.21, 0.35, 0.36-fold and ATP-dependent RNA helicase (DDX3X) downregulated by 0.36, 0.24, 0.27, 0.18, 0.27, 0.11-fold after treatment with SGL 04, SGL 07, SGL 10, SGL 12, SGL 15 and SGL 19, respectively.

We also evaluated the proteomics effects of LAB on keratinocytes pre-exposed to *S. aureus* (SA) and *S. pyogenes* (SP). First, we detected differentially expressed proteins in HaCaT cells treated with SA or SP as compared to control, identifying 241 and 224 deregulated proteins respectively (Supplemental Table 2). Among these, the up-regulated ones were highly associated with pathways that can suggest the pathogenic attack at molecular level, such as apoptosis, gene transcription and NOTCH4-related signaling pathways (Supplemental Table 4). Then we evaluated the proteomic effects of LAB on keratinocytes treated with SA or SP. As concerning keratinocytes exposed to SA, a total of 148, 209, 183, 135, 133, and 242 proteins were found differentially expressed after SGL 04, SGL 07, SGL 10, SGL 12, SGL 15 and SGL 19 treatments as compared to the untreated ones (Supplemental Table 2). Regarding, keratinocytes exposed to SP, a total of 224, 143, 123, 107, 145, 129, and 101 proteins were dysregulated after SGL 04, SGL 07, SGL 10, SGL 12, SGL 15 and SGL 19 treatments as compared to the untreated cells (Supplemental Table 2). To discern the functions of these dysregulated proteins a Reactome pathway enrichment analysis was performed for up and downregulated proteins (Supplemental Tables 4 and 5).

## Discussion

In the present study, with the aim of deepening the understanding of lactobacilli effects on skin cells, we carried out an analysis of keratinocytes treated with six specific LAB strains (*L. paracasei* SGL 04, *L. plantarum* SGL 07, *L. fermentum* SGL 10, *L. brevis* SGL 12, *L. casei* SGL 15 and *L. salivarius* SGL 19) to investigate their wound healing, anti-inflammatory, anti-pathogenic, and proteomics effects. A multidisciplinary approach, involving analytical chemistry, proteomics, biochemistry and microbiology, was applied to the analysis of HaCaT cells which represent a useful model to investigate repair response, anti-inflammatory interventions and infection of human keratinocytes^24^. This study was performed by treating HaCaT cells with LAB lysates since they offer a safer option than live bacteria for treatment of damaged skin^13,25^, and by using heat-killed pathogens since they still induce infection through their cell wall components^26^ assuring greater feasibility in the co-culture experiments.

Although live LAB, thanks to their active metabolism, have the advantage to produce potent antimicrobials (e.g., bacteriocins, organic acids, hydrogen peroxide) that could prevent adhesion and growth of some skin pathogens, they are generally not preferred in topical application, especially in situations where the skin barrier is breached^8,13^. In a wound situation, the potential risks of live LAB entering the bloodstream through breached skin have not been assessed, thus it is usually preferred to exploit the immunostimulating and anti-inflammatory power of lysates by giving up to antibacterial properties of live LAB^12^. Moreover, the lysates are more stable than viable cells at room temperature and are thus more suitable for topical applications^7,27^.

Here, we demonstrated that the six selected LAB lysates enhance the proliferation of keratinocytes, also after exposure to heat killed *S. aureus* or *S. pyogenes*, and that *L. plantarum* SGL 07 and *L. salivarius* SGL 19 lysates promote keratinocyte migration more than the others tested LAB. The data obtained have also shown that *L. plantarum* SGL 07 and *L. salivarius* SGL 19, improve the closure of wounds, a property which can therefore be linked to their induction effect of keratinocyte migration. The potential of *L. plantarum* lysate in aiding wound healing has been already showed for a specific strain called Lp-115^28^, while this is to the best of our knowledge the first time that the promotion of re-epithelialization is demonstrated for a *L. salivarius* lysate.

Interesting data, to increase the understanding of the beneficial effects of lactobacilli lysates on skin cells, were also suggested by the analysis of pro-inflammatory mediators performed by ELISA assays. Notably, keratinocytes produce various soluble factors such as chemokines and cytokines, which induce inflammation in response to a variety of stimuli, such as mechanical injury, allergens and bacteria^29^. The data obtained showed that all the LAB significantly reduced the release of the pro-inflammatory IL-8 from keratinocytes. This chemokine is produced by normal keratinocytes^30^ and its expression is induced in response to inflammatory stimuli and skin disease such as psoriasis^31^. We also demonstrated that the tested LAB lysates were able to decrease the secretion from HaCaT cells of other specific pro-inflammatory factors, such as MCP-1. It is known that the keratinocyte production of MCP-1 plays a key role in psoriasi^32^ and other skin disorders such as atopic and contact dermatitis^33,34^. Interestingly, we found that the secretion of MCP-1 from keratinocytes was induced after stimulation with heat-killed *S. pyogens*, but reverted and reduced by treatment with *L. paracasei* SGL 04, *L. plantarum* 07 and *L. salivarius* SGL 19 lysates. Also RANTES is a mediator which is over-secreted by keratinocytes in presence of psoriasis^35^ and skin disorders such as dermatitis^36^. We could also observe that the secretion of this inflammatory chemokine was reduced by keratinocytes treatments with *L. paracasei* SGL 04, *L. plantarum* SGL 07, *L. fermentum* SGL 10, and *L. brevis* SGL 12 lysates. These findings indicate that tested LAB lysates have different anti-inflammatory properties and suggest that they may find a use in fighting inflammatory skin diseases. Unfortunately, as concerning the anti-pathogenic effects of LAB lysates, competition assays revealed that at the dosage and timing tested they had no antimicrobial effects, even though viable *L. salivarius* SGL 19 and *L. fermentum* SGL 10 inhibited the growth of *S. aureus* and *S. pyogenes*, and viable *L. brevis* SGL 12 and *L. paracasei* SGL 04 inhibited *S. aureus* and *S. pyogenes*, respectively.

Nevertheless, the tested LAB lysates induced proteome modulations of the exposed keratinocytes. These modulations mainly involved the upregulation of proteins related to metabolism of RNA, amino acids and proteins, and migration (i.e. SLITs/ROBOs signaling^37^), as well as the downregulation of proteins related to inflammatory pathways. Indeed, in addition to interleukin-1, interleukins and cytokine signaling, proteins downregulated in HaCaT cells after LAB treatments were highly associated to NF-kB, Hedgehog, and RUNX pathways whose inactivation in HaCaT keratinocytes have been related to attenuated inflammatory cytokine expression and reduced skin inflammation^38–41^. Among the downregulated proteins involved in these processes we have identified for example ILF2 and DDX3X: reduced levels of ILF2 are implicated in lower transcription of the IL2^42^ and other cytokines^43^; whilst reduced levels of DDX3X modulate the NF-κB signal pathway and attenuate the production of inflammatory cytokines^44^.

Importantly, we also found that *L. plantarum* SGL 07 and *L. salivarius* SGL 19 lysates induced common proteins which may be related to their wound healing properties detected by scratch assay. The keratinocyte expression of envoplakin (EVPL) was induced 9.27-fold and 5.12-fold after SGL 07 and SGL 19 treatments, respectively. Envoplakin is involved in the dynamic interaction with periplakin and vimentin, facilitates the cytoskeletal reorganization and remodelling, and plays a key role in the wound healing process^45^. Furthermore, SGL 07 and SGL 19 lysates induced the keratinocyte expression of protein-arginine deiminase type-1 (PADI1), 7.22-fold and 4.58-fold respectively. PADI1, immunodetected in all the neo-epidermis, catalyzes the conversion of protein-bound arginines into citrullines, a deimination process which involves clot proteins and that is fundamental for the wound closure^46,47^. The results of migration assays indicated that the *L. plantarum* SGL 07 and *L. salivarius* SGL 19 lysates accelerate the wound closure likely by enhancing the movement of keratinocytes. However, considering that HaCaT cells have a doubling time ranging from 36 to 24 h in dependence of cell culture passages^48^, and that after 24 h there was no an increased migration, a mixed effect that also involves cell proliferation cannot be excluded after 7 days. Nevertheless, in support of a prevalent effect on migration, the proteomics data demonstrated that SGL 07 and SGL 19 lysates simultaneously induced (and to a greater extent than the other tested LAB) the expression of two proteins which play a key role in cellular migration: the focal adhesion kinase 1 (PTK2, also known as FAK) and the S100-A2 (S100A2) proteins. PTK2 was induced 6,68-fold and 3,26-fold whilst S100A2 2,47-fold and 2,15-fold after SGL 07 and SGL 19 treatments, respectively. PTK2, known since the 90s for promoting keratinocyte migration^49^, is a cytoplasmic protein tyrosine kinase considered a central molecule in integrin-mediated signalling involved in the regulation of actin cytoskeleton dynamics, and in the structure of cell adhesion sites and membrane protrusions which are important for cell movement^50^. Its inhibition reduces the keratinocyte migration especially under hypoxia. S100A2 is instead a calcium binding protein involved in cellular calcium signalling. It enhances cellular migration^51^, chemotaxis, and skin cancer metastasis^52^, by interacting with components of the motility apparatus such as tropomyosin and actin cytoskeleton^53^. Although mice expressing human S100A2 exhibited delayed wound repair^54^, it has been showed that S100A2 knockdown reduces the TGF-β1-induced cell migration^52^. Other proteins strongly induced in keratinocytes after treatment with SGL07, such as casein kinase II subunit alpha 3 and ribosome-binding protein 1, or after treatment with SGL19, such as actin aortic smooth muscle, are directly implicated in cell migration^55–57^. Proteomic analysis was also performed for keratinocytes exposed to pathogens and then treated with LAB lysates. This analysis revealed that the deregulated proteins of HaCaT cells exposed to *S. aureus*, or *S. pyogenes*, and then treated with LAB were highly associated with pathways suggesting beneficial effects of the treatments, such as for example the formation of the cornified cell envelope, the immune system, and neutrophil degranulation which were related to upregulated proteins, as well as apoptosis and DNA damage/telomere stress induced senescence which involved to the downregulated ones.

Our results support that LAB lysates have beneficial effect on the improving of skin health. We demonstrated wound healing and anti-inflammatory properties for the selected LAB lysates, and elucidated the molecular mechanism involved from a proteomic point of view. Further studies could be useful to deepen the knowledge of lactobacilli effects on skin cells, for example by investigating lysates at different concentrations or by analysing other different LAB strains. These strategies could also be useful to highlight antimicrobial effects of lysates that did not emerge from this study.

Furthermore, one of the important points highlighted by the present study is the strain specificity of the described effects. The six tested LAB lysates showed differences in the degree of stimulation of wound healing and proliferation of keratinocytes, as well differences in the induced modulation of keratinocytes proteome. Moreover, each strain showed a specific cytokine and chemokine secretion profile which may reflect different anti-inflammatory or immunomodulatory effects. These findings suggest that formulations containing a combination of different LAB strains could be potentially more effective than those containing single strains. However, multi strain combinations should be investigated for their modes of action, their interaction (antagonistic or synergistic), in order to find the best combination and the appropriate concentration to target specific skin disorders such as allergic dermatitis, acne, wounds, psoriasis, photoaging and atopic dermatitis.

Further investigations in suitable animal models of skin disorders should be performed prior to proceeding to clinical trials in humans in order to confirm the efficacy and safety of these formulation in prevention and therapy of skin disorders.

## Supplementary information

Supplementary Figures.

Supplementary Table 1.

Supplementary Table 2.

Supplementary Table 3.

Supplementary Table 4.

Supplementary Table 5.

## Data Availability

Data are available via ProteomeXchange with identifier PXD018088, username: reviewer01067@ebi.ac.uk, password: 5QJfEx7e.

## Abbreviations

LAB: Lactic acid bacteria
TNF-α: Tumor necrosis factor-alpha
IFN-γ: Interferon-gamma
PBS: Phosphate buffered saline
DMEM: Dulbecco’s modified Eagle's medium
MCP-1: Monocyte chemoattractant protein-1
RANTES: Regulated on activation, normal T cell expressed and secreted
LC–MS/MS: Liquid chromatography–tandem mass spectrometry
SWATH: Sequential window acquisition of all theoretical mass spectra
