# Supplementary material for: Exploring the wound healing, anti-inflammatory, anti-pathogenic and proteomic effects of lactic acid bacteria on keratinocytes
Source: Sci Rep. 2020 Jul 14;10:11572. doi: 10.1038/s41598-020-68483-4 (PMC7360600; doi:10.1038/s41598-020-68483-4)
Supplement: Supplementary file 1 — Supplementary Figures. [file 41598_2020_68483_MOESM1_ESM.pdf]

Exploring the Wound Healing, Anti-Inflammatory, Anti-Pathogenic and Proteomic Effects of Lactic Acid Bacteria on Keratinocytes

Jessica Brandi, Samuele Cheri, Marcello Manfredi, Claudia Di Carlo, Virginia Vita Vanella, Federica Federici, Eleonora Bombiero, Alda Bazaj, Eleonora Rizzi, Laura Manna, Giuseppe Cornaglia, Umberto Marini, Maria Teresa Valenti, Emilio Marengo, Daniela Cecconi

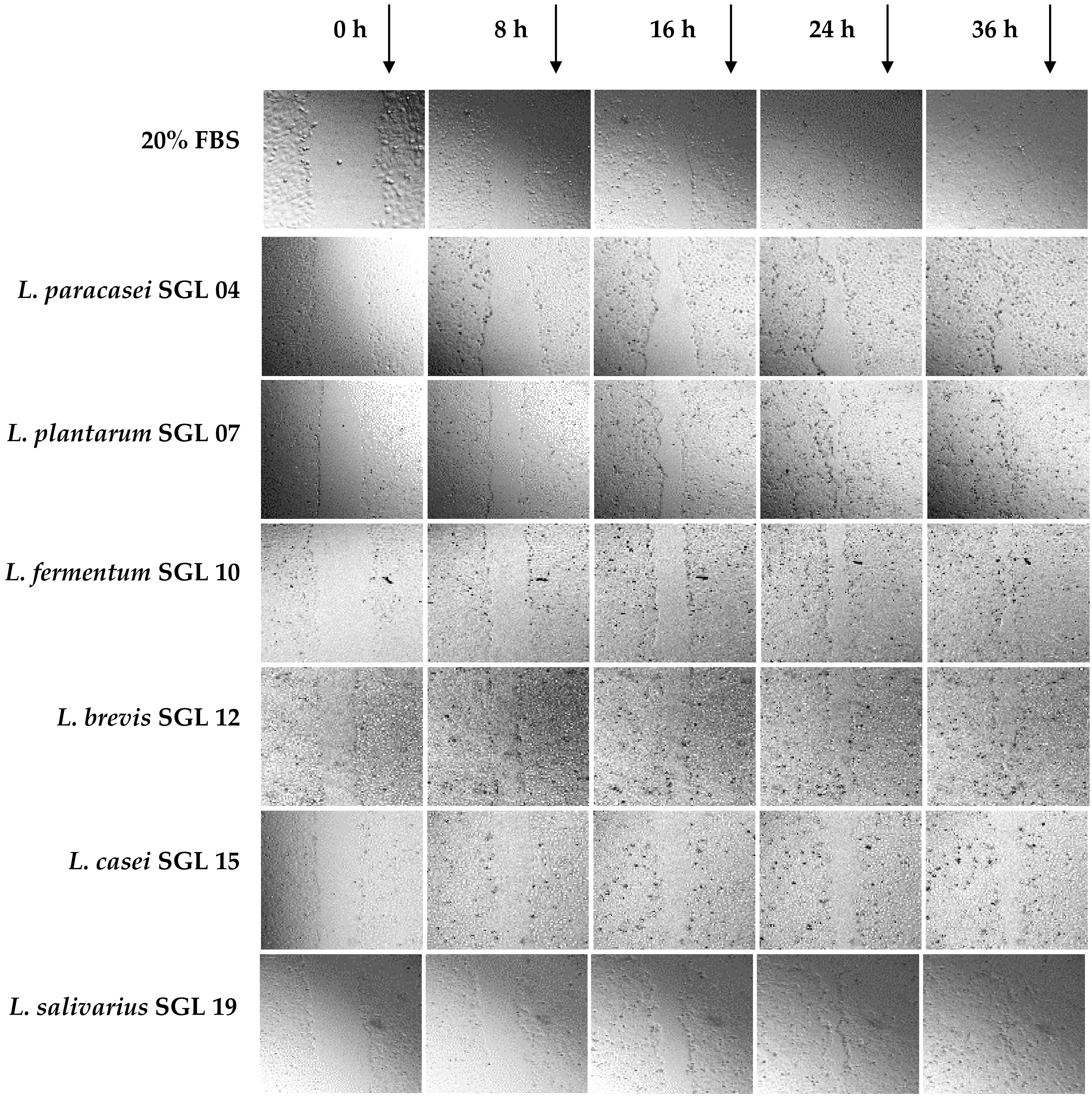

Supplemental Figure 1. Representative images (magnification, x200) of wound closure at 0, 8, 16, 24 and 36 h in the presence of positive control (20% FBS) or LAB lysates.

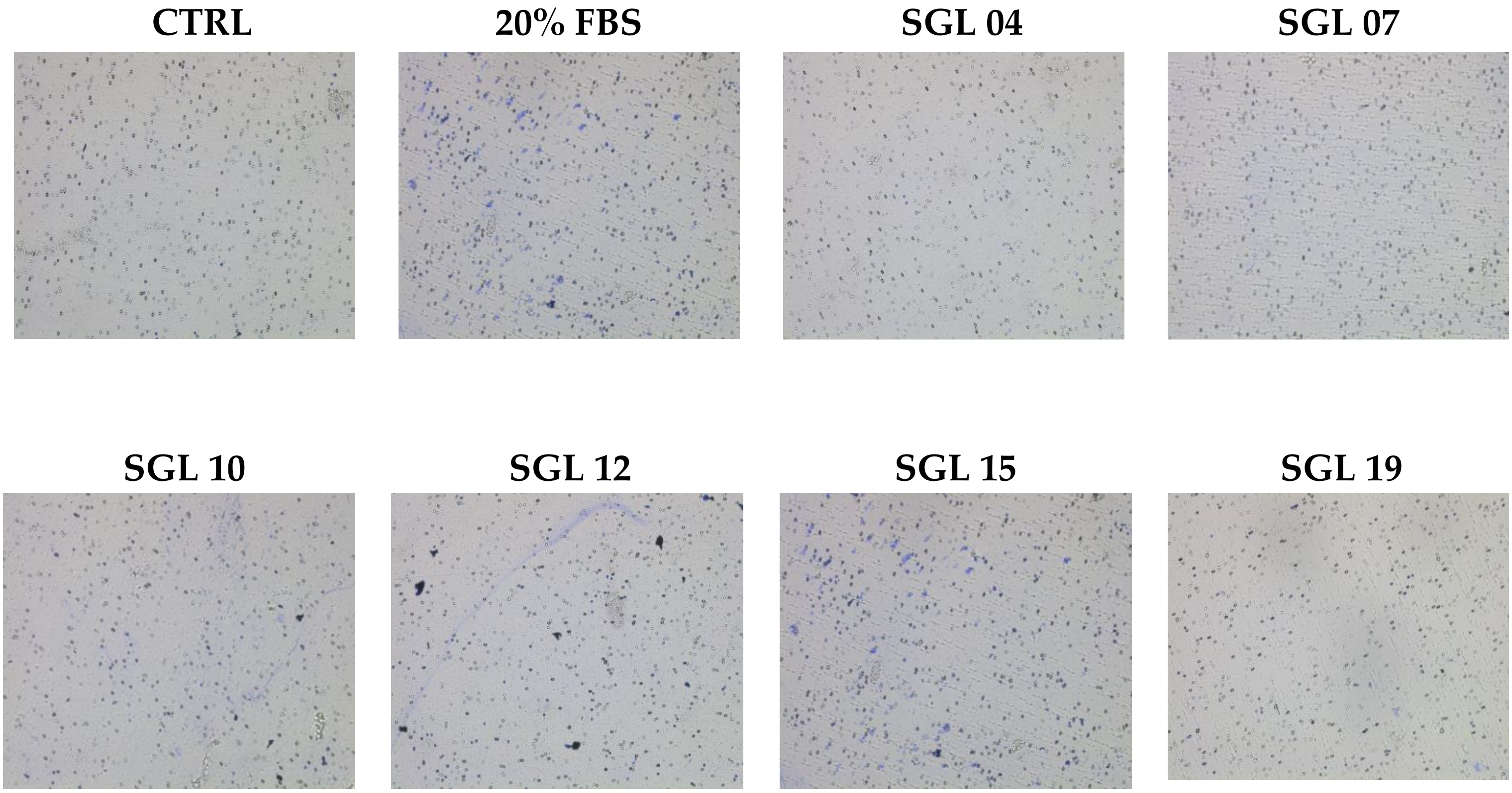

Supplemental Figure 2. Representative images of the transwell migration assay after 24h in untreated control HaCaT cells (CTRL), positive control (20% FBS) and upon treatment of keratinocytes with LAB lysates (magnification 20x).

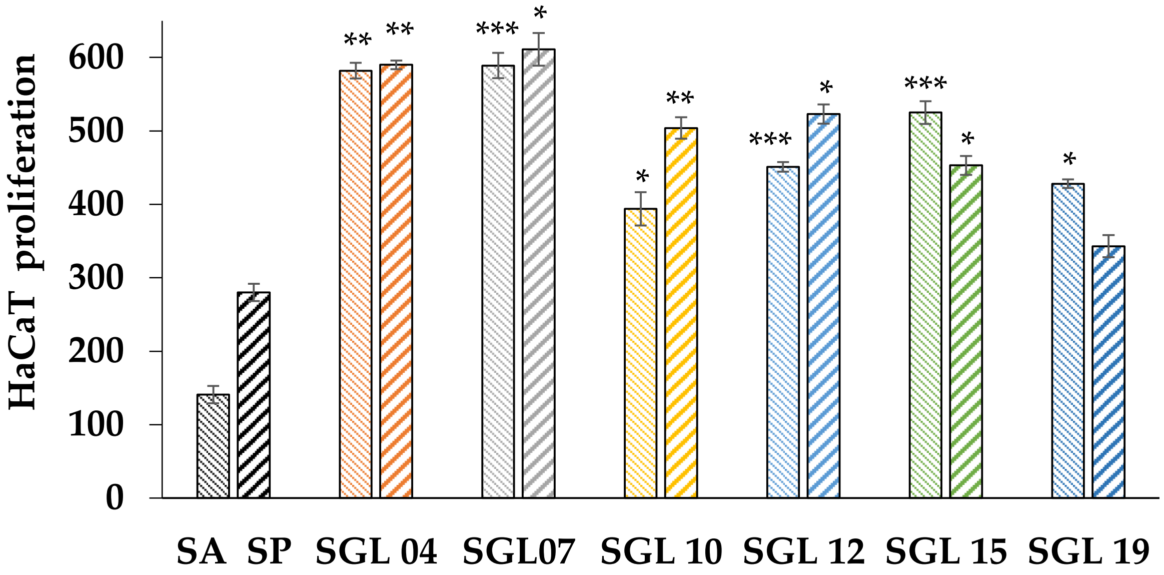

Supplemental Figure 3. Proliferation of keratinocytes after treatment for 12h with *S. aureus* (SA) or *S. pyogenes* (SP), and exposure for 12h to LABs, measure by MTS reduction (% of control, i.e. untreated HaCaT cells). Light and dark gray histograms indicate HaCaT cells treated with SA and SP, respectively. Results are expressed as the mean ± SEM, ****$p < 0.0001$, ***$p < 0.001$, **$p < 0.01$, *$p < 0.05$.

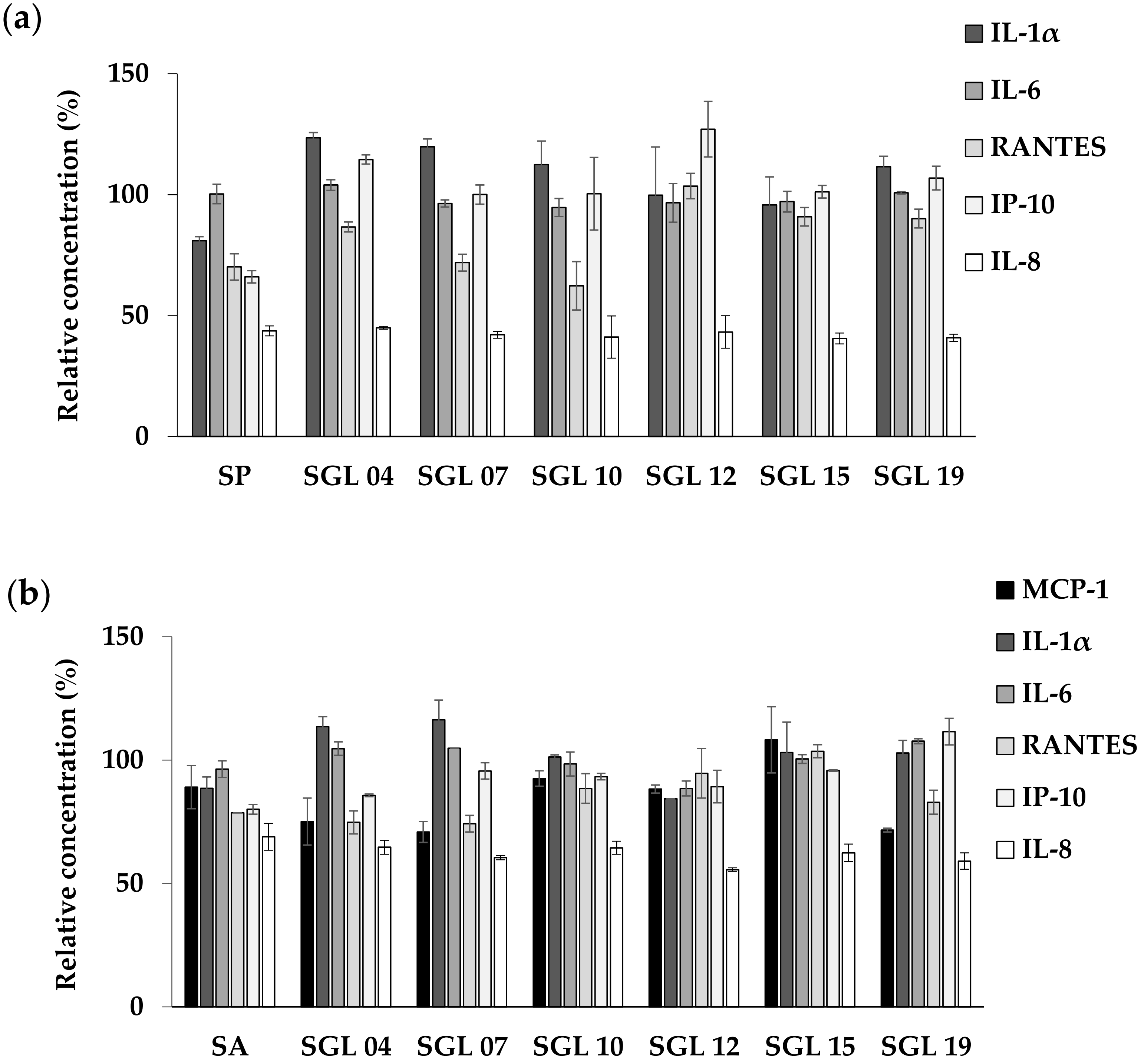

Supplemental Figure 4. Chemokines and cytokines secretion from HaCaT cells subjected to stimulation with (**a**) *S. pyogenes* (SP) or (**b**) *S. aureus* (SA) and then exposed to the different LAB lysates. Relative concentration (%) was determined by dividing the average concentration of chemokine or cytokine in LAB-treated HaCaT cells by concentration in control (untreated HaCaT cells) × 100. Results are expressed as the mean ± SEM.
